# Hypersensitivity and the Working Environment for Allergy Nurses in Sweden

**DOI:** 10.1155/2014/681934

**Published:** 2014-04-06

**Authors:** Pia Kalm-Stephens, Therese Sterner, Kerstin Kronholm Diab, Greta Smedje

**Affiliations:** ^1^Department of Women's and Children's Health, Uppsala University, 751 85 Uppsala, Sweden; ^2^Division of Woman, Child and Reproduction, Department of Pediatrics, Skåne University Hospital, 205 02 Lund, Sweden; ^3^Occupational and Environmental Medicine, University and Regional Laboratories Region Skåne, 205 02 Lund, Sweden; ^4^Department of Medical Sciences/Occupational and Environmental Medicine, Uppsala University, 751 85 Uppsala, Sweden

## Abstract

*Background*. Allergy nurses are exposed to allergens and respiratory irritants, and there are no national guidelines addressing personnel safety when working with these agents. *Objective*. To investigate the prevalence of allergies, asthma, and hypersensitivity symptoms among allergy nurses and the use of protective equipment and measures when working with allergen concentrates and respiratory irritants. *Methods*. A questionnaire survey was performed among the members of the Swedish Association of Allergy Nurses. *Results*. Diagnosed asthma was reported by 17%, while 18% had allergy to pets, 28% had allergy to pollens, and 26% reported nasal symptoms. Fifty-one percent reported a history of asthma, allergic diseases, or hypersensitivity symptoms in their family. Exhaust ventilation was used by 24% during skin prick tests, 17% during allergen specific immunotherapy, and 33% when performing methacholine challenge tests. Tightly closed containers for disposable waste were used by 58% during skin prick tests, by 60% during immunotherapy, and by 40% during Pc provocation tests. *Conclusion*. Allergy nurses had a tendency to increased prevalence of lower respiratory symptoms, asthma, and allergic rhinitis and more than half of the nurses had a family history of asthma, allergic diseases, or hypersensitivity symptoms. Additional studies are needed to evaluate the validity of these results.

## 1. Introduction

Allergy nurses work with various tests aimed at diagnosing allergic diseases and other hypersensitivity disorders. Common tests are skin prick test (SPT), where different allergens are used, penicillin (Pc) provocation tests, and tests with various respiratory irritants, such as methacholine (MCH). Many allergy nurses also perform allergen specific immunotherapy (ASIT) by injecting the allergen concentrates. In the clinical working situation with the patients, it is common to be exposed to drops of allergen extracts and to dried allergen on extract bottles and tissues used when performing SPT and ASIT. All these exposures may potentially lead to effects on the health of the nurses. Efforts have been made to improve the working environment in some clinics, using fume or downdraft hoods for diagnostic tests, whereas such preventive and safety measures are missing in other clinics. There are no existing national guidelines for these specific tests concerning safety for the allergy nurses and local recommendations may vary.

Four main risk factors have been identified for developing occupational asthma (OA): the causative factor of exposure to an agent at work, the predisposing factors of atopy and genetic predisposition, and the contributing factor of cigarette smoking. Rhinoconjunctivitis is more likely to appear before the onset of IgE associated OA, and IgE sensitization and OA are most likely to develop in the first years of exposure [1].

Within the Swedish Association of Allergy Nurses (ASTA), discussions have been carried out concerning deficiencies in the working environment and there have been reports of members that have become sensitized or developed respiratory symptoms at work but there are few studies on this issue. Previously, there was a case report concerning two nurses in Sweden, who often conducted MCH tests and who developed an increased bronchial hyperreactivity related to their work [2]. In 1991, ASTA conducted a questionnaire survey among all members, studying the correlation between asthma and bronchial provocation tests. Among the 259 (80%) respondents, 27% were working with MCH and 15% with histamine tests. Among those who performed the MCH tests, 31% reported respiratory symptoms associated with the test situation and 4% (3 subjects) reported the development of asthma during the time they had been working with such tests [3]. In cooperation with the Department of Occupational Medicine in Gothenburg, Sweden, further analysis was carried out. No evidence for a relationship between MCH tests and development of new asthma was found, but it was concluded that individuals with bronchial hyperreactivity could experience respiratory problems associated with testing, especially if they did not use respiratory protection [4].

There are a few international studies that indicate higher frequencies of asthma and bronchial hyperreactivity among “respiratory therapists (RT)” [5, 6]. An RT works to prevent, treat, and assist in the diagnosis of respiratory diseases and asthma is one of several assignments for the RT. The studies indicate that cleaning of instruments, such as bronchoscopes, in glutaraldehyde and administration of certain antiviral agents in aerosol form are the main risk factors [7, 8]. Furthermore, a higher incidence of asthma among nurses has been reported in some studies [9–12]. The principal risks recognized are the handling of detergents and disinfectants, the use of latex gloves, and the administration of certain drugs in aerosol form. All these studies are only partially relevant for nurses working in Sweden with asthma and allergy, since their main exposures are allergen extracts and respiratory irritants when performing, for example, SPT, ASIT, and MCH provocation tests and specific safety guidelines concerning work exposure to allergens, except for a recently published case report describing a compounding technician, with a known allergy to timothy grass, who experienced anaphylaxis after a needle stick from a stock bottle of timothy grass extract while preparing immunotherapy vials [13]. The authors of this case report established that this raises a new question of occupational safety for allergy immunotherapy extract-compounding personnel safety not previously discussed in the allergy literature. They also considered a need for preemployment and regular screening for allergen sensitivity in these employees. In February 2010, ASTA organized a workshop on this subject and it was decided to perform a questionnaire survey among its members for the purpose of gathering information about these issues as a primary orientation to further discussions.

### 1.1. Study Objectives

The aims of the current study were to

- investigate the prevalence of allergies, asthma, and hypersensitivity symptoms among the members of ASTA;
- investigate the use of protective equipment and measures when working with allergen concentrates and respiratory irritants;
- gather data for further discussion concerning the working environment.

## 2. Methods

Data were collected using a web-based questionnaire that contained 40 questions addressing personal characteristics, work situation, protection at work, and hypersensitivity symptoms. The questions on work situation and protection at work were constructed by the project group, while questions on hypersensitivity symptoms were taken from validated questionnaires, particularly those used in the European Community Respiratory Health Survey (ECRHS) II [14]. Furthermore, one question concerned heredity (parents or siblings) for asthma and allergies. All questions were presented with fixed alternative answers. The questions used in the questionnaire to evaluate hypersensitivity symptoms are presented in Table 1. Similar symptoms were compiled in groups and these group definitions (Table 1) are used in the presentation of the results.

ASTA is an organization with members from most parts of Sweden and represents a great majority of nurses working with asthma and allergy in Sweden. It includes nurses working both in primary care and in hospitals.

Webropol, a web-based survey tool provided by the County Council of Uppsala, Sweden, was used to gather the data. An e-mail was sent out in February 2011 to all ASTA members containing information about the study and a link to the survey. A paper questionnaire was provided to a few members without an e-mail address. In both cases, three reminders were sent out. After the data collection was completed, an Excel file with all the data was compiled and the data were subsequently deleted from the Webropol server. The study was approved by the Regional Ethical Review Board in Uppsala, Sweden.

Most of the data are descriptive in character, since the purpose of the study was to describe certain aspects of allergy nurses′ health and working environment. The alternative answers to the questions concerning frequencies of performing SPT, Pc, and MCH provocation tests were classified into five groups: never, less than once a week, 1–5 times a week, 6–10 times a week, and more than 10 times a week. Data were compiled into two groups: ≤5  times a week and >5 times a week in order to identify nurses who had low or high exposure to these agents and to establish larger groups. Subjects that had been working with specific tasks for different periods of time (0–2 years, 3–5 years, 6–10 years, and more than 10 years) were also compiled into two groups: nurses who had worked ≤5  years and >5 years, for the same reasons as mentioned above. Five years was chosen as suitable cut-off time to be able to include possible new onset asthma, allergies, and hypersensitivity symptoms that would appear after some years in the profession. Differences in symptoms between groups were analyzed by *χ* ^2^ Test, and a significance level of 5%.

There was no control group in this study but symptom prevalences among the allergy nurses were compared with data on women aged 30–67 years old from the general population collected by the Swedish part (Stockholm, Gothenburg, Umeå, and Uppsala) of the GA2LEN survey [15].

## 3. Results

The questionnaire was sent to 585 nurses belonging to ASTA: 570 via e-mail and 15 via paper mail. After three reminders, 418 had answered, resulting in a participation rate of 71%. The average age of responders was 53 years (range 30–67 years) and 98% were female. 71% reported that they had never smoked regularly, 28% were previously smokers, and 0.5% were current smokers.

**Figure 1:**
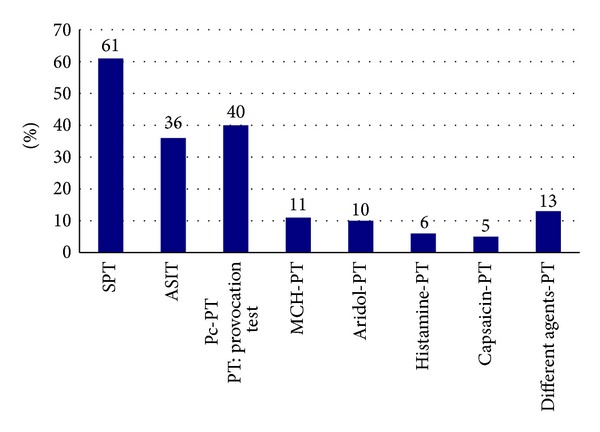
Specific working tasks of nurses in ASTA (%); *n* = 256.

Fifty-eight percent of the responders worked in primary care, 41% in hospitals, and 1% in both. Thirty-four percent worked with children, 37% with adults, and 29% with both children and adults. Forty-four percent worked with asthma and allergy approximately one day a week and 27% worked two to four days a week while 29% worked almost exclusively with asthma and allergy. A majority, 56%, had worked with asthma and allergy for more than ten years, and 75% for more than five years. Among the requested tasks, SPT was the most common task performed, followed by Pc provocation tests and ASIT (Figure 1). MCH provocation test was the most common test among various hyperreactivity provocations, being performed by 11% (Figure 1).

### 3.1. Symptoms

Asthma diagnosed by a doctor was reported by 17% of the responders to the questionnaire, while 18% had allergy to furry pets and 28% to pollens and 31% reported allergic rhinitis. Thirty-four percent reported bronchial hyperreactivity to cigarette smoke, fumes, strong odors, and so forth. Lower respiratory symptoms in the last 12 months, when not having a cold, were reported by in total 48%. Symptoms of wheezing were reported by 21%, shortness of breath by 7%, and dry cough at night by 24%. Itchy rash was reported by 15%, while 26% had nasal symptoms such as sneezing or a runny or a blocked nose and itchy eyes without having a cold or the flu. Fifty-one percent acknowledged that they had a history of asthma, allergic diseases, or hypersensitivity symptoms in their family.

#### 3.1.1. Work-Related Symptoms

Thirty-three members (8%) reported that they had asthma, allergies, or hypersensitivity symptoms related to their working situation. Of these, asthma or some kind of respiratory symptoms was reported by 28 subjects. Twenty-one subjects reported allergic rhinitis and 25 nurses were bothered by nasal symptoms. Two subjects had been out of work for more than ten days during the last six months due to colds or bronchial problems. Of the 28 nurses who reported work-related asthma or some kind of respiratory symptoms, 19 developed these symptoms after entering the profession. Two-thirds of the nurses, who had reported work-related symptoms, had a family history of asthma, allergic diseases, or hypersensitivity disorders.

All ASTA members were also asked about when their hyperreactivity, respiratory, or asthma symptoms first appeared. Fifty-seven percent of the responders reported that the symptoms occurred after some years in the profession, while 43% indicated that they had their symptoms before entering the profession. In the case of allergic rhinitis or nasal symptoms, 37% reported no symptoms prior to the work as an allergy nurse. The same results were seen in the group that reported work-related eczema.

### 3.2. Diagnostic Tests

#### 3.2.1. Skin Prick Test and Symptoms

**Figure 2:**
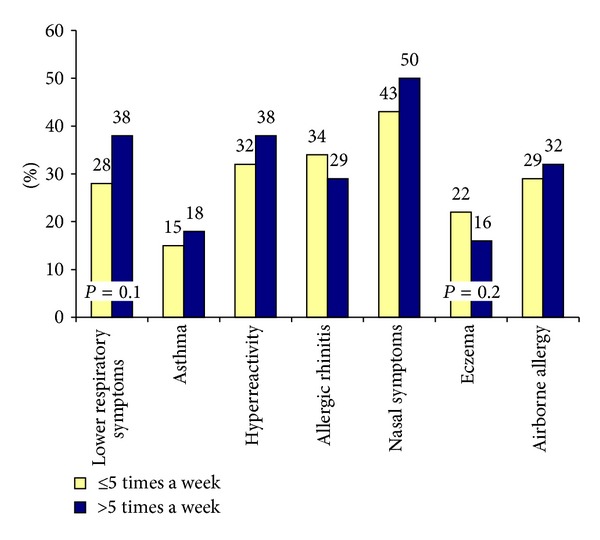
Prevalence (%) of symptoms in nurses who have worked with SPT ≤5 times a week and >5 times a week; *n* = 256.

Lower respiratory symptoms during the last 12 months were reported by 38% of those who performed SPT more than five times a week compared with 28% for those who did fewer tests although not significant in *χ* ^2^ Test (Figure 2). However, there was a tendency to reverse results regarding allergic rhinitis, where 29% of those who performed SPT more than five times a week reported symptoms, compared with 34% for those who worked with SPT less frequently, *P* = 0.3 in the *χ* ^2^ Test.

**Figure 3:**
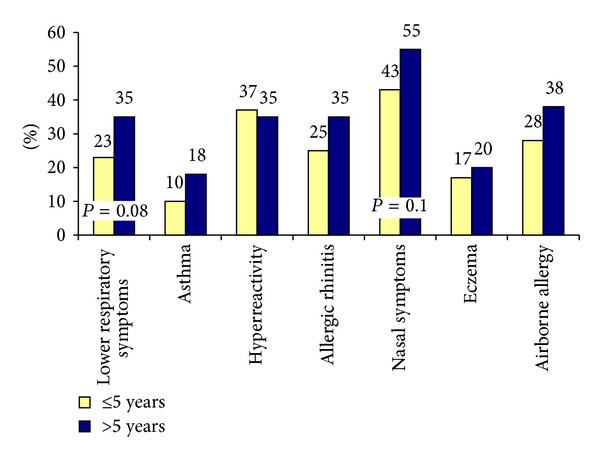
Prevalence (%) of symptoms in nurses who have worked with SPT for ≤5 years and for >5  years; *n* = 256.

There was a tendency that the nurses in ASTA who had worked with SPT for more than five years, being 65% of those who performed SPT, experienced more symptoms than those who had worked for a shorter period, although the differences were not statistically significant in the *χ* ^2^ Test (Figure 3).

#### 3.2.2. Pc Provocation Test and Symptoms

**Figure 4:**
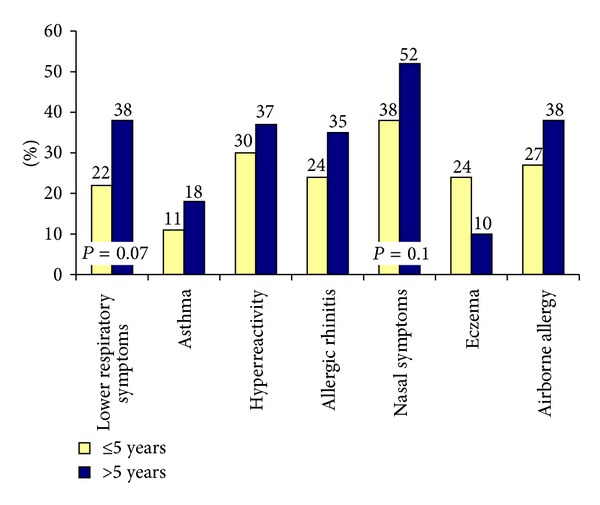
Prevalence (%) of symptoms in nurses who have worked with Pc provocations for ≤5 years and for >5 years; *n* = 157.

Pc provocations were performed by 40% of the respondents and 59% of these reported some kind of symptoms. There was no difference in the prevalence of lower respiratory symptoms in nurses who performed Pc tests less than once a week compared with those who worked with this test one to five times a week. Nobody worked with Pc provocation tests more than 5 times a week. However, there was a tendency that those who had performed Pc provocation tests for more than five years reported more lower respiratory symptoms, allergic rhinitis, nasal symptoms, and airborne allergy, although the differences were not statistically significant in the *χ* ^2^ Test (Figure 4).

#### 3.2.3. MCH Provocation Test and Symptoms

**Figure 5:**
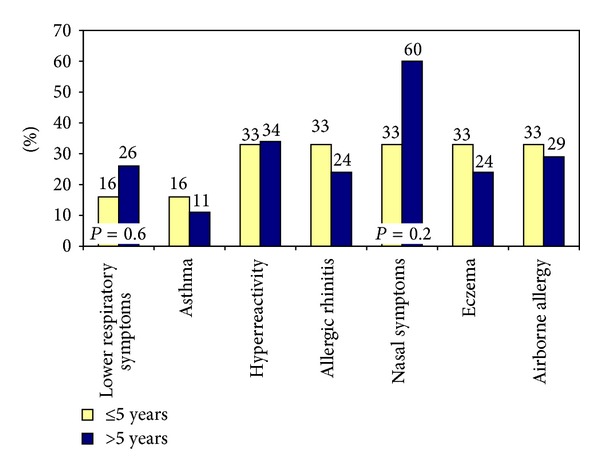
Prevalence (%) of symptoms in nurses who have worked with MCH provocations for ≤5 years and for >5 years; *n* = 44.

MCH provocation tests were performed by 44 (11%) of the ASTA members and 25 performed this task more than once a week. In the latter group, 10 nurses reported that they had asthma, hyperreactive airways, or lower respiratory symptoms and for seven subjects the symptoms had developed after they had started work as allergy nurses. Nasal symptoms were reported by 14 of the nurses who performed this test more than once a week (25) and 8 reported allergic rhinitis. Eighty percent of these nurses reported either airway or nasal symptoms, or both. A large majority, 38 of the 44 nurses who performed MCH provocation tests, had worked with this test for more than 5 years. Nasal and lower respiratory symptoms are common among nurses who have worked with MCH provocation test for more than five years but due to few nurses in the groups, the differences are not statistically significant in the *χ* ^2^ Test (Figure 5).

### 3.3. Protective Measures

**Figure 6:**
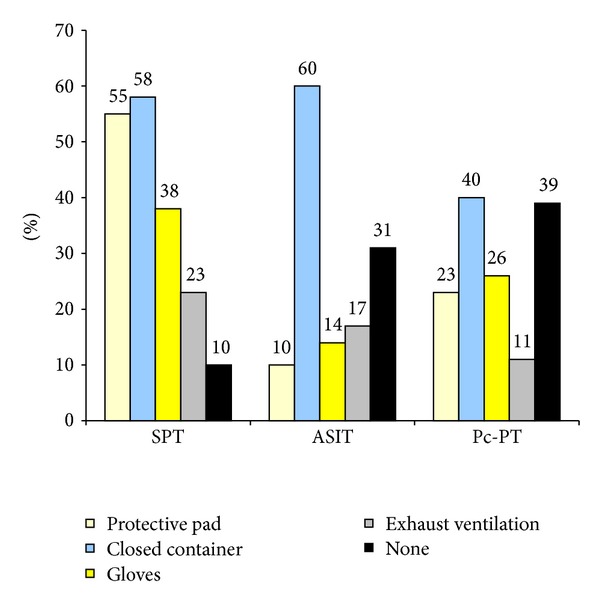
Protective equipment used during different work tasks (%); *n* = 256.

The use of protective equipment during SPT, ASIT, and Pc provocation tests varied, as is shown in Figure 6. A closed container is a container with a lid aimed to stop evaporation from used materials containing allergens and respiratory irritants, and a protective pad is a paper mat used to create a clean working area. Approximately, a third of the responders did not take any protective measures when performing ASIT and Pc provocation tests. Protective equipment during MCH provocation tests was primarily aimed at the presence of respiratory irritants. Most of the responders, (73%) used a separate room or space with additional ventilation and the remainder mainly used the fume hood or downdraft hood. Thirteen percent used gloves and 4% respiratory protection devices. For MCH provocation tests, nobody reported that no protective measures were taken.

## 4. Discussion

**Figure 7:**
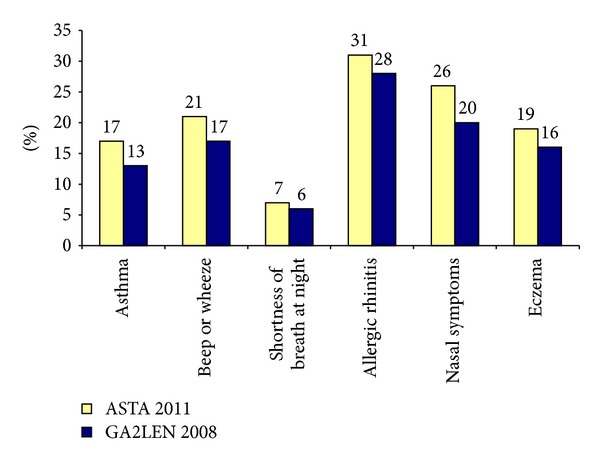
Prevalence of asthma and respiratory symptoms in ASTA's members and in the Swedish part of the GA2LEN study; *n* = 14000.

The results show that asthma and hypersensitivity symptoms are common among the members of ASTA, that most of the subjects have been in the profession for a long time, and that over half of the nurses have a family history of asthma, allergy, or hypersensitivity symptoms. In order to make a preliminary evaluation of the results concerning asthma allergies and hypersensitivity symptoms, a comparison has been done with the prevalence in the Swedish general population [14, 16]. A general population is judged to be neutral and unexposed to these agents and working tasks. Since almost all respondents in the present ASTA survey were women, a comparison with data from the Swedish part of the GA2LEN survey for women, from 2008, was chosen to be the most suitable [15]. The prevalence among ASTA's members seems to be slightly higher for most symptoms (Figure 7).

About one-third of the responders to the questionnaire reported that they become hyperreactive in contact with cigarette smoke and so forth. Another Swedish survey on the general population showed a prevalence among women of the same magnitude, 31% [17].

The high proportion of allergy nurses with a family history of asthma, allergy, or hypersensitivity symptoms suggests that there might be a biased selection of individuals entering the profession. If this is the case, it is very important that the working environment is adapted to their special needs. There are regulations to protect employees in the working environment. In addition to the general Work Environment Act [18], the Swedish Work Environment Authority has also issued provisions on Systematic Work Environment Management 2001:01 [19], Workplace Design, AFS 2009:02 [20], and the Chemical Working Environment, AFS 2011:19 [21]. The employer is responsible for establishing a working environment that does not expose workers to health risks and to investigate the working environment regularly to assess such risks. For example, hazardous chemicals that may occur at work should be identified and working conditions at such sites need to be planned to minimize the exposure. The working premises should have ventilation so that air quality in the breathing zone is satisfactory and air pollution generated in the operations should be effectively removed. However, the rules are very general. Exposures, such as allergens and substances for respiratory provocations, which are relevant to allergy nurses, are not discussed explicitly. Furthermore, the professionals in health care have established recommendations for performing these tasks, but there appear to be few recommendations in such methodology manuals and so forth concerning how these substances should be handled from an occupational perspective.

The present survey found that the use of protective measures varied between different working tasks. The proportion of lack of use of any protection was the highest (39%) for those working with Pc provocations, compared with 10% for those working with SPT. There was also a higher proportion who reported symptoms among those who carried out the Pc provocation tests (59%), compared with that for SPT (52%). The highest risk for symptoms (80%) seemed to be caused by performing MCH tests more regularly, even though protection measures, in many cases, had been used. The reason why nurses have not used protection measures might be linked to the fact that occupational safety for allergy nurses has not been discussed in a sufficient way, neither in the clinical settings nor in the scientific literature.

Health problems linked to the working situation cannot have been a major concern for a majority of the ASTA members, since 56% had been in the profession for ten years or more. It may be difficult to establish a connection between work and perceived symptoms when the symptoms are the same as those perceived by the general population. The specific knowledge owned by allergy nurses may have had an impact on their assessment of their own hyperreactive symptoms. However, 8% considered that they had developed allergies or hypersensitivity disorders as a result of their work and 4.5% (19 nurses) reported that their respiratory problems had started after initiating work with allergens or provocations. The number of new cases of asthma among women in the general population has been estimated to be approximately 1.8/1000 person-years and the number of new cases of rhinitis to be approximately 7/1000 person-years [22, 23]. On the basis of these figures, 0.7 new cases of asthma and 2.8 new cases of rhinitis per year could be predicted in a group of 400 women. We do not know exactly for how long time the nurses had worked with asthma and allergy, but more than half of them had worked for more than 10 years. If we assume an average experience of 15 years, the number of new cases of asthma would be 11, while at 20 years of experience it would be 14 new cases. The corresponding figures for rhinitis would be 42 or 56 new cases. Based on these conceptual calculations, the number of responders, who reported that they had new hypersensitivity symptoms which they attribute to their work, is not significantly higher than the incidence of new symptoms in the general population. Nevertheless, the frequency of those reporting current symptoms was higher than in the general population. Furthermore, we found an increase in symptoms among those having performed provocation tests for a longer period.

### 4.1. Method Discussion

The validated questions taken from ECHRS II have been suitable for the purpose of this study, but the questions on work tasks that were constructed for this study could have been more specific. The classification concerning frequencies of work tasks was somewhat arbitrary and might have influenced the outcome of the results. However, when comparing symptoms in groups with different number of years in service, the use of different cut-points gave similar results (a tendency to increased symptoms with numbers of years in service). The participation rate was 71% which should be satisfactory but only 29% of the respondents worked exclusively with asthma and allergy and 44% worked just one day a week. Consequently, a large proportion of the studied group had a regular but not a high exposure to allergens and respiratory irritants. There might also be a “healthy worker effect,” since the study was only directed towards present members of ASTA, thus not giving any information from nurses who have left the profession and ASTA. However, the purpose of the study was to give some general descriptive information of an issue which has not been previously highlighted.

## 5. Conclusions

Among the members of ASTA, there is a tendency towards increased prevalence of lower respiratory symptoms, asthma, allergic rhinitis, nasal symptoms, and eczema and more than half of the nurses had a family history of these diseases and symptoms. With this knowledge, it is of great importance to be observant of the work environment and of hypersensitivity disorders in the nurses. Preventive work environment efforts have largely been made concerning MCH provocations. The results of this study imply that further work should also be carried out to establish recommendations concerning protective measures for allergen management and other tasks. Additional studies are needed to evaluate the validity of these results and to be able to answer more specific questions concerning causal relationship between the working conditions and the occurrence of respiratory and allergic symptoms.

## Figures and Tables

**Table 1:** Questions used for evaluating hypersensitivity symptoms and definitions used in the presentation of the results.

Questions	Definitions
Did you have wheezing or whistling in chest at any time in the last 12 months? Have you been woken up by an attack of shortness of breath at any time in the last 12 months? Did you have dry cough at night without having a cold or upper respiratory infection in the last 12 months?	Lower respiratory symptoms

Do you have a doctor diagnosed asthma?	Asthma

Do you get hyperreactive airways in contact with cigarette smoke, fumes, strong odours, and so forth?	Hyperreactivity

Do you have any nasal allergies, including hay fever?	Allergic rhinitis

Have you ever had a problem with sneezing or a runny or a blocked nose when you did not have a cold or the flu? Has this nose problem been accompanied by itchy eyes?	Nasal symptoms

Have you ever had an itchy rash that was coming and going for at least 6 months? Have you had this itchy rash in the last 12 months?	Eczema

Do you have allergies to furry pets? Do you have allergies to pollens? Do you have allergies to dust mites? Do you have allergies to mold?	Airborne allergy
